# CaDrA: A Computational Framework for Performing Candidate Driver Analyses Using Genomic Features

**DOI:** 10.3389/fgene.2019.00121

**Published:** 2019-02-19

**Authors:** Vinay K. Kartha, Paola Sebastiani, Joseph G. Kern, Liye Zhang, Xaralabos Varelas, Stefano Monti

**Affiliations:** ^1^Bioinformatics Program, Boston University, Boston, MA, United States; ^2^Section of Computational Biomedicine, Boston University School of Medicine, Boston, MA, United States; ^3^Department of Biostatistics, Boston University School of Public Health, Boston, MA, United States; ^4^Department of Biochemistry, Boston University School of Medicine, Boston, MA, United States; ^5^School of Life Sciences and Technology, ShanghaiTech University, Shanghai, China

**Keywords:** oncogenic driver analysis, stepwise search, TCGA, CCLE, R package

## Abstract

The identification of genetic alteration combinations as drivers of a given phenotypic outcome, such as drug sensitivity, gene or protein expression, and pathway activity, is a challenging task that is essential to gaining new biological insights and to discovering therapeutic targets. Existing methods designed to predict complementary drivers of such outcomes lack analytical flexibility, including the support for joint analyses of multiple genomic alteration types, such as somatic mutations and copy number alterations, multiple scoring functions, and rigorous significance and reproducibility testing procedures. To address these limitations, we developed Candidate Driver Analysis or CaDrA, an integrative framework that implements a step-wise heuristic search approach to identify functionally relevant subsets of genomic features that, together, are maximally associated with a specific outcome of interest. We show CaDrA’s overall high sensitivity and specificity for typically sized multi-omic datasets using simulated data, and demonstrate CaDrA’s ability to identify known mutations linked with sensitivity of cancer cells to drug treatment using data from the Cancer Cell Line Encyclopedia (CCLE). We further apply CaDrA to identify novel regulators of oncogenic activity mediated by Hippo signaling pathway effectors YAP and TAZ in primary breast cancer tumors using data from The Cancer Genome Atlas (TCGA), which we functionally validate *in vitro*. Finally, we use pan-cancer TCGA protein expression data to show the high reproducibility of CaDrA’s search procedure. Collectively, this work demonstrates the utility of our framework for supporting the fast querying of large, publicly available multi-omics datasets, including but not limited to TCGA and CCLE, for potential drivers of a given target profile of interest.

## Introduction

Advances in high-throughput sequencing technology has led to a rapid rise in the availability of large multi-omic datasets through compendia such as the CCLE, TCGA, the Genotype-Tissue Expression (GTEx), and others (Barretina et al., 2012; Chang et al., 2013; Ardlie et al., 2015). These data include genetic alterations, comprising SCNAs and somatic mutations, epigenetic information, such as microRNA expression and DNA methylation, as well as gene expression profiling through microarray or RNA-sequencing (RNASeq) technology, across tens of thousands of samples representing varying biological contexts. Concomitantly, several computational methods have been developed and applied to effectively query and integrate different types of genome-wide datasets in order to make meaningful predictions about the biological processes driving the phenotypes of interest (Drier et al., 2013; Kristensen et al., 2014). An important application of such methods is the identification of recurrent genomic alterations, and their potential effects on downstream pathway activity or phenotypes associated with development and disease states. For example, in many cancers, samples exhibiting elevated activity of a given oncogenic signature may be enriched for, or driven by functionally relevant somatic mutations or SCNAs. Identifying such associations may help elucidate underlying mechanisms contributing to abnormal pathway activity, further enabling disease subtyping and sample classification (Bea et al., 2005; Savage et al., 2003; Monti et al., 2012). Alternatively, linking these genomic features with their close interactors through protein-protein interaction networks, gene function annotations or phenotypic readouts such as drug sensitivity may support the discovery of novel druggable targets and further guide precision medicine regimens (Bild et al., 2006; Heiser et al., 2011; Daemen et al., 2013; Hou and Ma, 2014; Jia and Zhao, 2014).

Recently, computational methods and models have been developed for performing driver gene analyses applied to high-dimensional ‘omics’ data from cancer cell lines and patients. These are typically motivated either by frequency or exclusivity of alterations across samples (Youn and Simon, 2011; Ciriello et al., 2012; Dees et al., 2012; Vandin et al., 2012; Lawrence et al., 2013; Leiserson et al., 2013; Kim et al., 2016), or their functional interplay based on biological interaction networks and pathway ontology (Ng et al., 2012; Creixell et al., 2015; Leiserson et al., 2015; Cho et al., 2016). Indeed, certain approaches integrate interactome and functional information to further guide driver gene prioritization in cancer (Chen et al., 2014; Xi et al., 2017; Sanchez-Vega et al., 2018). Some of these tools have been proposed to specifically identify subsets or combinations of genomic features that are collectively associated with a given phenotypic response, explaining a larger fraction of the biological context than any individual feature alone (Kim et al., 2016). These methods, while useful, do not offer simultaneous support for: (i) the joint analyses of multi-type features, including SCNAs and somatic mutations, with possible extension to other genomic data, (ii) multiple feature scoring functions and, most importantly, (iii) rigorous assessment of the statistical significance of the discovered associations. Of equal relevance, a user-friendly and flexible programming package supporting the rapid screening for candidate drivers given a set of ranked genomic features is currently lacking, and would prove extremely useful for incorporation in analytical pipelines aimed at the generation of novel biological hypotheses.

Here, we present CaDrA, a methodology that searches for the set of genomic alterations, here denoted as *features* (mutations, SCNAs, translocations, etc.), associated with a user-provided ranking of samples within a dataset. Our method specifically employs a stepwise heuristic search to identify a subset of features whose union is maximally associated with the observed sample ranking, and carries out rigorous statistical significance testing based on sample permutation, thereby allowing for the identification of candidate genetic drivers associated with aberrant pathway activity or drug sensitivity, while still exploiting aspects of feature complementarity and sample heterogeneity. To highlight the method’s overall performance, along with its relevance and ability to select sets of genomic features that indeed drive certain oncogenic phenotypes in cancer, we perform extensive evaluation of CaDrA based on simulated data, as well as real genomic data from cancer cell lines and primary human tumors. The results from simulations show that CaDrA has high sensitivity for mid- to large-sized datasets, and high specificity for all sample sizes considered. Using genomic data drawn from CCLE and TCGA, we demonstrate CaDrA’s capacity to correctly identify well-characterized driver mutations in cancer cell lines and primary tumors spanning multiple cancer types, along with its ability to discover novel features associated with invasive phenotypes in human breast cancer samples, which we functionally validate *in vitro*. Our framework, which is publicly available as an R package, will allow for rapidly mining numerous multi-omics datasets for candidate drivers of user-specified molecular readouts, such as pathway activity, drug sensitivity, protein expression, or other quantitative measurements of interest, further enabling targeted queries and novel hypothesis generation.

## Results

### CaDrA Overview

**FIGURE 1:**
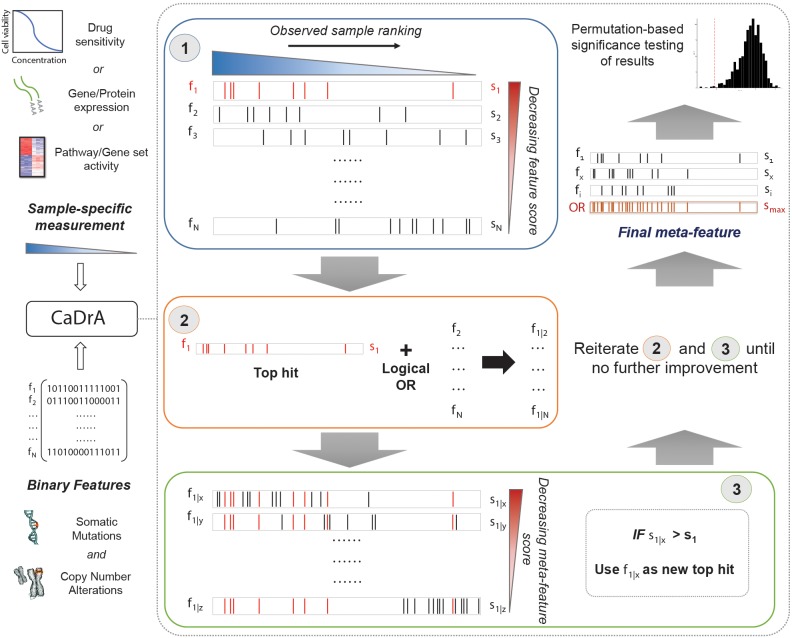
Overview of CaDrA workflow and implementation. CaDrA takes as input a sample-specific measurement to rank samples, and a matrix of binary features of the same samples. In Step 1 (blue box), CaDrA begins by choosing a starting feature, which is either the single feature having the best score based on its left-skewness, or a user-specified start feature. In the next step (Step 2; orange box), the union (logical OR) of this feature with each of the remaining features in the dataset is taken, yielding ‘meta-features’ with their corresponding scores. If any meta-feature has a better score than the hit from the previous step (Step 3; green box), CaDrA uses this new meta-feature as a reference for the next iteration, repeating Steps 2 and 3 until no further improvement in scores can be obtained. The final output is a set of features (meta-feature) whose union has the (local) maximum score and its permutation-based *p*-value.

An overview of CaDrA’s workflow is summarized in Figure 1. CaDrA implements a step-wise heuristic approach that searches through a set of binary features [each represented as a 1/0-valued vector, indicating the presence/absence of a SCNA, somatic mutation, or other (epi)genetic alterations across samples, respectively], and returns a final subset of features whose union (logical OR) defines an alteration ‘meta-feature’ that is maximally associated with the defined sample ranking provided as input (see section “Methods”). The strength of the association of a meta-feature with a sample ranking is a function of the agreement between the skewness of the alterations’ occurrences and the sample ranking. The input sample ranking is usually a function of a sample-specific measurement, e.g., the activity level of a pathway, the response to a targeted treatment, the expression level of a given transcript or protein, etc. Therefore, the meta-feature returned by the search is the set of features maximally predictive of that same sample-specific measurement variable. The logical OR operator used in the iterative search framework specifically takes advantage of heterogeneity seen across samples (i.e., samples harboring similar phenotypes but different drivers of the given outcome), thus enabling the potential identification of complementary drivers of target phenotypes (Kim et al., 2016). CaDrA allows for multiple modes to query ranked binary datasets with user-specified parameters defining search criteria, enables rigorous permutation-based significance testing of results, and reduced computation time by exploiting pre-computed score distributions and parallel computing, when available (see section “Methods”).

### Analysis of Simulated Data to Evaluate CaDrA Performance

**FIGURE 2:**
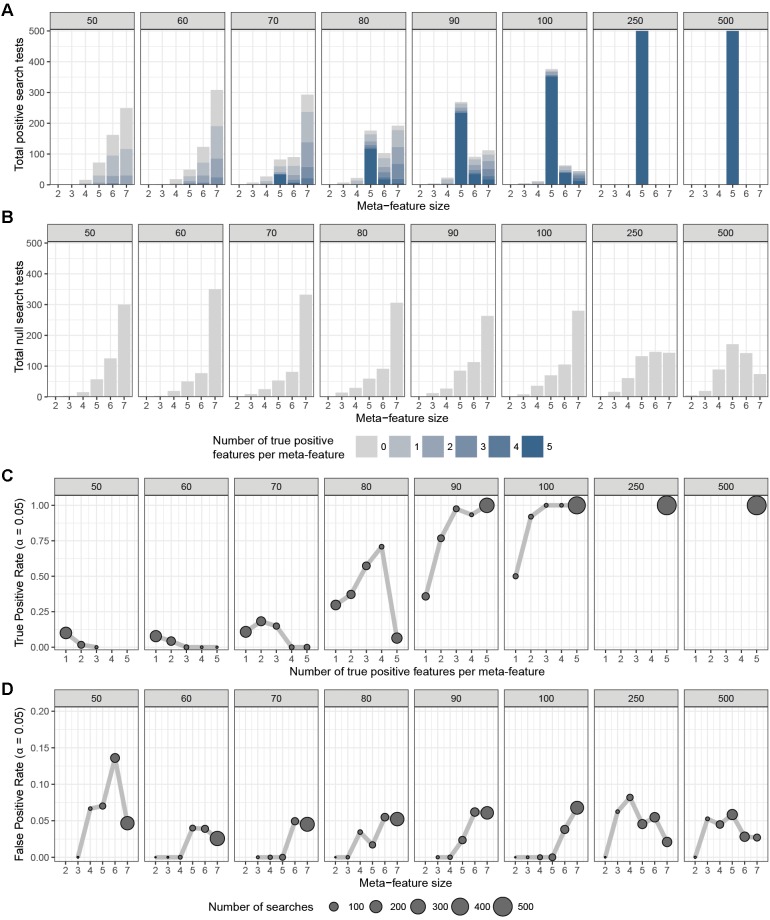
CaDrA performance on simulated data. CaDrA was run on 500 independent simulated datasets containing **(A)** both positive and null, and **(B)** only null features with sample sizes ranging between 50 and 500 samples (number in gray box above each sub-panel). In each case, the distribution of the number of features per meta-feature (i.e., the meta-feature size) returned by CaDrA is shown **(A,B)** as well as the number and fraction of searches that yielded significance for α = 0.05 **(C,D)**, corresponding to the true positive rate (TPR) and false positive rate (FPR), respectively.

To assess the overall performance of CaDrA to recover (statistically) significantly associated meta-features, we simulated two types of datasets for a range of sample sizes: (i) the *true-positive datasets* consist of both left-skewed (i.e., true positive with skewness concordant with sample ranking) as well as uniformly distributed (i.e., null) features; and (ii) the *null datasets* consist of null features only (see section “Methods” and Supplementary Figure S1). This enabled us to estimate the overall sensitivity and specificity of CaDrA using the true positive and null datasets, respectively. By running CaDrA on multiple simulated datasets of different sample sizes (*n* = 500 true positive and null datasets for each sample size), we first evaluated the resulting meta-features based on the number of true positive features and the total number of features contained within each returned meta-feature (i.e., the meta-feature size; Figure 2A,B). The true positive datasets had a maximum of five positive features to be detected, while the maximum number of features CaDrA was allowed to add was set to 7, to evaluate the ability of the search to recover all but no more than the positive features. With progressively higher sample sizes, we observed an increase in the fraction of CaDrA-identified meta-features that include all 5 true positive features (Figure 2A). The TPR and FPR of CaDrA on the simulated positive and null data, respectively, for different sample sizes are shown in Figure 2C,D, and was calculated as the fraction of searches returning meta-features with permutation *p*-value significant at α = 0.05 (Supplementary Figure S2). The TPR was estimated for different numbers of recovered true positive features (in the true positive datasets), while the FPR was estimated for different numbers of returned features (by definition, false positives) in the null datasets, and is summarized in Table 1. CaDrA returned all of the simulated true positive features with 100% TPR for sample sizes larger than *N* = 100. CaDrA also yielded a very high mean TPR of >95% at *N* = 100, with the sensitivity dropping to 7.7% only at the smallest sample size of *N* = 50 (Table 1). Further, when applied to the null datasets (Figure 2B), the majority of meta-features returned by CaDrA were correctly deemed as non-significant at α = 0.05, with a maximum mean FPR of 7.2% for the lowest sample size analyzed (Figure 2D and Table 1).

**Table 1:** Overall true positive rate (TPR) and false positive rate (FPR) of CaDrA based on simulated data.

Sample Size (*N*)	Mean TPR (%)	Mean FPR (%)
50	7.69	7.2
60	5.76	2.8
70	11.53	3.8
80	30.72	4.6
90	87.55	5
100	96.51	4.6
250	100	4.6
500	100	4.2


**Weight-averaged TPR and FPRs were computed per sample size for true positive and null simulated datasets, respectively (n = 500 simulated datasets per sample size; see section “Methods”)*.*

These results suggest that CaDrA requires mid- to large-sized datasets for sufficient sensitivity, while maintaining high specificity at all sample sizes assessed.

### CaDrA Identifies Known Regulators of Ras/Raf/Mek/ERK Signaling Sensitivity in Cancer Cell Lines

The mitogen-activated protein kinase (MAPK) kinase (MEKK)/extra-cellular signal-regulated kinase (ERK) pathway is a well-conserved kinase cascade known to play a regulatory role in cell proliferation, differentiation, and survival in response to extracellular signaling (Kim and Choi, 2010; Cargnello and Roux, 2011; Burotto et al., 2014). Increased MAP/ERK kinase (MEK) activity is a feature of many cancers, and is often triggered by missense mutations in *BRAF* and *NRAS*, two upstream oncogenes and potent regulators of Ras/Raf/Mek/ERK signaling (Cantwell-Dorris et al., 2011; Burotto et al., 2014). Small molecules targeting these mutated proteins have been shown to be effective in treating these cancers via inactivation of Ras/Raf/Mek/ERK signaling (Roberts and Der, 2007; Chapman et al., 2011; Barretina et al., 2012; Johnson and Puzanov, 2015). To highlight CaDrA’s ability to recover independent genomic features that may confer hypersensitivity of cancer cells to targeted small molecule treatment, we utilized drug sensitivity profiles for MEK inhibitor AZD6244 (Yeh et al., 2007), along with matched genomic data from CCLE. Specifically, we used per-sample estimates of ‘ActArea’ or area under the fitted dose response curve, a metric that has been shown to accurately capture drug response behavior (Jang et al., 2014), to rank cell lines from high to low sensitivity, as well as data comprising somatic mutations and SCNAs as the binary feature matrix (see section “Methods”). CaDrA was then run to look for a subset of features associated with increased sensitivity to treatment with AZD6244 (i.e., increased ActArea scores).

**FIGURE 3:**
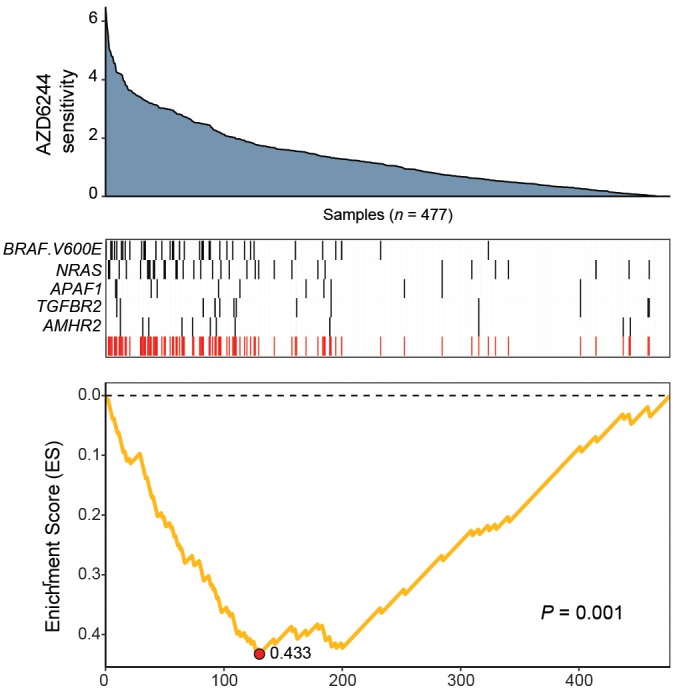
CaDrA identifies mutations in MAPK/ErK signaling genes that contribute to hyper-sensitivity to MEK inhibition *in vitro*. ActArea measurements reflecting sensitivity to MEK inhibitor AZD6244 were used to rank CCLE cell lines (*n* = 477). CaDrA was then run to identify sets of genomic features that were most-associated with decreasing ActArea (i.e., increasing sensitivity) scores. Through step-wise search iterations, CaDrA identified somatic mutations in known regulators upstream of MEK, including an activating mutation in *BRAF* (BRAF^*V*600*E*^) and *NRAS*, as well as those in *APAF1*, *TGFBR2*, and *AMHR2*, before terminating the search process. The resulting meta-feature (red track) and its corresponding enrichment score (ES) is shown.

The resulting feature set (i.e., meta-feature) is shown in Figure 3. Remarkably, CaDrA selected the BRAF^*V*600*E*^ and *NRAS* somatic mutations in the first two iterations, respectively. Subsequent iterations identified mutations in *APAF1*, *TGFBR2*, and *AMHR2*, before terminating the search process (*P* ≤ 0.001). APAF1 is a pro-apoptotic factor and known regulator of cell survival and tumor development (Ferraro et al., 2003), the depleted expression of which has been observed in malignant melanoma cell lines and specimens (Soengas et al., 2006). TGFBR2 and AMHR2 are both type II receptors functioning as part of the transforming growth factor (TGF)/bone morphogenetic protein (BMP) superfamily, together serving as mediators of cellular differentiation, proliferation and survival, and play important roles in directing epithelial-mesenchymal transition (EMT) (Rojas et al., 2009; Stone et al., 2016). Notably, MAPK signaling activity can also be regulated by TGF/BMP stimulation (Derynck and Zhang, 2003; Moustakas and Heldin, 2005; Chapnick et al., 2011), suggesting that these mutations are potential independent drivers of increased MEK signaling, and hence, of increased sensitivity to treatment with AZD6244. We next extended our analysis of cancer cell line sensitivity profiles to alternative small molecules targeting MEK (PD-0325901), as well as RAF (PLX4720 and RAF265). The meta-features associated with increased sensitivity to each of the four drug treatments assessed are shown in Supplementary Figure S3 and summarized in Table 2. Importantly, both BRAF^*V*600*E*^ and NRAS mutations were identified as candidate drivers of sensitivity to MEK inhibition by AZD6244 and PD-0325901. Furthermore, the BRAF^*V*600*E*^ mutation was returned by CaDrA for all four independent queries, highlighting its association with increased sensitivity to inhibitors targeting the same protein (BRAF) as well as its downstream effector (MEK).

**Table 2:** Summary of mutation subsets identified by CaDrA as associated with elevated Mek and Raf inhibition in cancer cell lines.

Target	Treatment	CaDrA hits	*P*-value
MEK	AZD6244	*BRAF.V600E, NRAS, APAF1, TGFBR2, AMHR2*	0.001
MEK	PD-0325901	*BRAF.V600E, NRAS, TRIM33*	0.001
RAF	PLX4720	*BRAF.V600E*	0.001
RAF	RAF265	*TTK, BRAF.V600E, ZMYM2, IL21R, BCL11B, MAP3K5, TAF15*	0.005


**Mutation meta-features identified as associated with increased sensitivity to inhibitors targeting Mek (AZD6244, PD-0325901) and Raf (PLX4720) are shown, along with the corresponding permutation* p-value of each search result.*

Collectively, these results confirm CaDrA’s capability to accurately identify upstream drivers of cellular response to treatment that are both components of independently linked pathways, as well as part of the same signaling branch, which in turn suggests their role in driving the disease state of interest.

### CaDrA Identifies Hallmark Drivers Associated With Protein Biomarkers in Human Cancers

Protein abundance levels have widely been utilized to histologically classify several human tumor subtypes, with relevant diagnostic and therapeutic implications. Epidermal Growth Factor Receptor (EGFR) expression, for instance, together with *EGFR* mutation status can be used to predict response to existing anti-EGFR treatments in patients with lung cancers (Pao et al., 2004; Mascaux et al., 2011). To demonstrate CaDrA’s targeted search mode when identifying genomic alterations that track with a pre-defined starting feature, we ran CaDrA using phosphorylated EGFR (EGFR^Tyr1068^) protein expression levels to stratify TCGA lung adenocarcinomas (LUAD), and seeded the search process with EGFR mutations. Subsequent search iterations selected well-known regulators of EGFR activity in lung cancers, including mutations in epithelial-to-mesenchymal transition mediators *SMAD4* and *LAMC2*, as well as *ERBB2* (Liu et al., 2015; Moon et al., 2015), with the meta-feature being statistically significant based on the permuted null background obtained for the same search criterion (*P* ≤ 0.02; Supplementary Figure S4).

**FIGURE 4:**
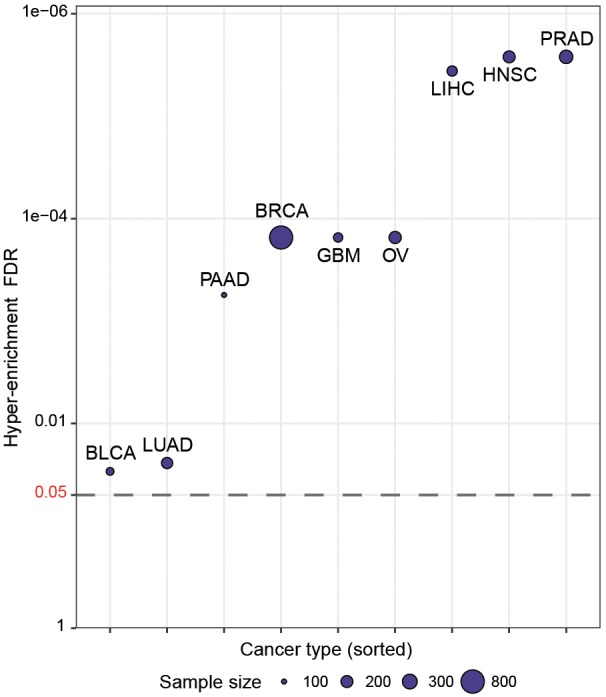
CaDrA systematically identifies known drivers of onco-proteins and tumor suppressor proteins in human cancers. TCGA genomic data for nine different cancer types were queried using the expression of distinct proteins mapping to hallmark genes included in COSMIC (*n* = 57) for sample ranking. Resulting meta-features identified by CaDrA were then pooled across all protein queries and tested for enrichment against a reference COSMIC-defined gene list (*n* = 554). FDR-adjusted gene set enrichment *p*-values are shown, with cancer types sorted in decreasing order of FDR *q*-value. BLCA, bladder urothelial carcinoma; BRCA, breast invasive carcinomas; GBM, glioblastoma multiforme; HNSC, head and neck squamous cell carcinoma; LIHC, liver hepatocellular carcinoma; LUAD, lung adenocarcinoma; OV, ovarian serous cystadenocarcinoma; PAAD, pancreatic adenocarcinoma; PRAD, prostate adenocarcinoma. Points are plotted in -log_10_ space.

We then wished to more systematically determine whether CaDrA can identify known drivers of target profiles previously associated with oncogenic and tumor-suppressive markers in human cancers. To do so, we queried TCGA expression profiles of proteins encoded by a set of hallmark genes that are defined in the COSMIC database (Forbes et al., 2017), along with genomic data from nine different cancer types in TCGA (Forbes et al., 2017). Briefly, for each cancer type, a CaDrA query was performed with respect to each of the proteins corresponding to the COSMIC-defined oncogenes or tumor suppressor genes (*n* = 57). In particular, CaDrA was applied to search for sets of genomic features associated with elevated protein expression for each protein under consideration. The features selected by CaDrA were then pooled across all protein queries, and the resulting feature set was tested for enrichment against the reference COSMIC list of frequently mutated oncogenes and tumor suppressor genes (*n* = 554; see section “Methods”). We observed a significant enrichment of the reference cancer driver mutations among the CaDrA-identified features in all cancer types tested (Hyper-enrichment FDR < 0.05; Figure 4 and Supplementary Table S1). These results validate CaDrA’s ability to identify independently cataloged, functionally relevant genomic drivers in primary human malignancies.

### CaDrA Reveals Novel Drivers of Oncogenic YAP/TAZ Activity in Human Breast Cancer

**FIGURE 5:**
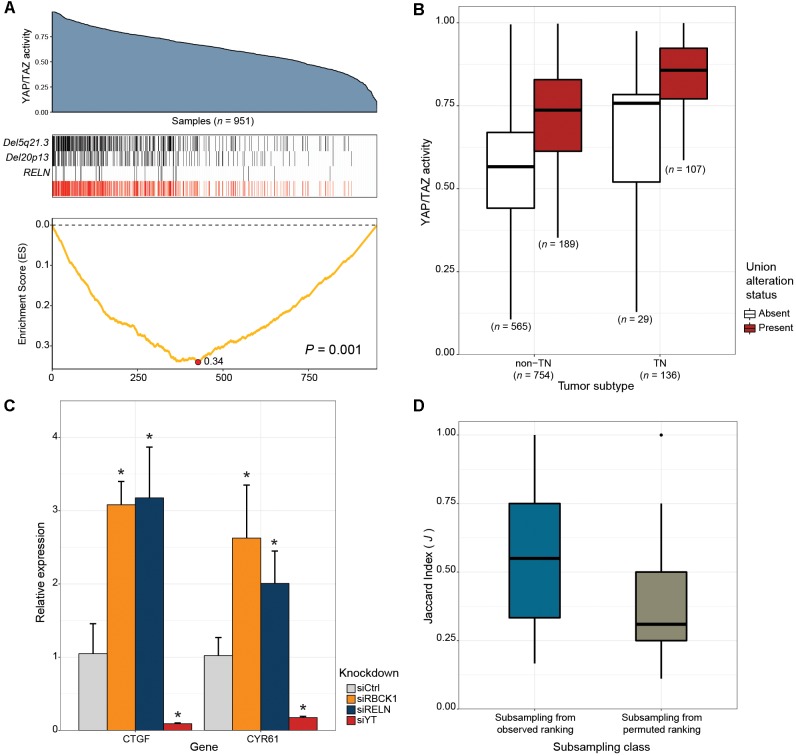
CaDrA identifies novel drivers of oncogenic YAP/TAZ activity in human breast carcinomas. **(A)** TCGA BRCA RNASeq data (*n* = 951) was projected onto the space of YAP/TAZ-activating genes (blue area plot; see section “Methods”). A CaDrA search for features associated with elevated YAP/TAZ activity identified two chromosomal deletions (*Del5q21.3*, *Del20p13*), and a somatic mutation in *RELN* (black tracks). The union of the three features (red track) and the corresponding running enrichment score (ES) is also shown. **(B)** Box plot of YAP/TAZ activity estimates for triple negative (TN) and non-TN TCGA BRCA samples. Sample groups are further stratified by the presence or absence of the union alteration status of the meta-feature identified by CaDrA (panel a, red track). Only samples with known TN status were considered **(C)** siRNA-mediated knockdown of 20p13-harboring gene *RBCK1*, and *RELN* in HS578T cells resulted in significant increase in the expression levels of canonical YAP/TAZ targets CTGF and CYR61, as indicated by their relative qRT-PCR expression, confirming the identified CaDrA hits as potential regulators of BRCA-associated YAP/TAZ activity. **(D)** Sub-sampling-based reproducibility assessment for candidate drivers of YAP/TAZ activity compared to a CaDrA query for a random profile ranking in TCGA BRCAs. Jaccard (*J*) indices of the returned meta-features obtained with and without sub-sampling (repeated for *n* = 100 independent sub-sampling iterations) were computed and compared for the two queries, yielding a significantly higher *J* index distribution for the original query relative to the permuted ranking query (Wilcox *P* < 0.0001). Ctrl: Scrambled control; YT: YAP/TAZ; ^∗^ FDR < 0.05; two-tailed Student’s *t*-test.

Next, we tested whether our framework can be applied to the discovery of novel drivers of oncogenic pathways in cancer. The Hippo signaling pathway is a highly conserved developmental pathway known to play an essential role in cell proliferation and survival (Varelas, 2014). YAP (Sudol, 1994), and TAZ (Kanai et al., 2000) serve as central downstream transcriptional effectors of the pathway. Aberrant nuclear YAP/TAZ localization and transcriptional activity is associated with a range of cancers, including BRCAs (Hiemer et al., 2015; Moroishi et al., 2015; Zanconato et al., 2015, 2016). To identify alternative genetic events that can potentially explain the elevated YAP/TAZ activity exhibited in some human breast cancers, we applied CaDrA using genomic data from the TCGA BRCA sample cohort, along with corresponding per-sample estimates of YAP/TAZ activity derived using a gene expression signature of YAP/TAZ knockdown in MDA-MB-231 cells (see section “Methods”). Samples with available RNASeq, somatic mutation and SCNA profiles (*n* = 957) were first ranked in decreasing order of their overall YAP/TAZ activity estimates. The ranked binary matrix of mutation and SCNA features were then used as input to CaDrA. In the first iteration, CaDrA identified the top scoring genomic feature to be a deletion on chromosomal locus chr5q21.3 (Figure 5A), harboring tyrosine kinase receptor-encoding gene *EFNA5*. *EFNA5*, a member of the Eph receptor family, has been hypothesized to function as a tumor suppressor, whose expression has been shown to be reduced in human BRCAs relative to normal epithelial tissue (Fu et al., 2010). Advancing to a second iteration, CaDrA then identified an additional deletion of chr20p13 as the next-best feature (Figure 5A). The chr20p13 genomic deletion spans multiple genes (Supplementary Table S2), including *RBCK1*, whose reduced expression has been shown to be associated with increased tumor cell proliferation and survival, as well as with poor prognosis in breast cancer (Donley et al., 2014). CaDrA then proceeded to identify somatic mutations in the *RELN* gene, before terminating the search process (*P* ≤ 0.001; Figure 5A). Loss of *RELN* expression has indeed been shown to induce cell migration in esophageal carcinoma, and to be associated with poor prognosis in breast cancer (Stein et al., 2010; Yuan et al., 2012). To ensure that the derived meta-feature association is not a spurious consequence of correlation with tumor subtype, we tested for the association of YAP/TAZ activity with the meta-feature while controlling for BRCA TN status using a linear regression model. The results confirmed that the positive association between YAP/TAZ activity and the occurrence of these genomic alterations is independent of BRCA patho-histology (linear regression meta-feature coefficient *P* < 0.0001; Figure 5B). Analysis of YAP/TAZ activity based on the same knockdown signature in CCLE BRCA cell lines (*n* = 59; Supplementary Figure S5A) shows that *RBCK1* and *RELN* display the highest anti-correlation between their gene expression and YAP/TAZ activity (Supplementary Figure S5B). In order to assess whether these identified candidates indeed drive the elevated YAP/TAZ activity phenotype, we performed siRNA-mediated knockdown of *RELN* or *RBCK1* in HS578T breast cancer cells, followed by expression quantification of YAP/TAZ canonical targets, which serves as a read-out of nuclear YAP/TAZ activity (Piccolo et al., 2014). HS578T cells which, similar to MDA-MB-231 cells from which the gene signature was derived, are TN BRCA cells but display lower overall YAP/TAZ activity (rank 7/59) compared to the latter (rank 54/59). Importantly, knockdown of either of these candidate drivers in these cells yielded a significant increase in expression levels of YAP/TAZ targets CTGF and CYR61 (FDR < 0.05; two-tailed Student’s *t*-test), validating the association of their loss of function with increased YAP/TAZ transcriptional activity (Figure 5C).

Thus, application of CaDrA to the analysis of YAP/TAZ activity in primary BRCA samples identified multiple new candidate drivers, with *in vitro* validation confirming the causal role of the top two candidates, *RBCK1* and *RELN*, in driving this activity. These results highlight our tool’s ability to discover novel oncogenic genomic drivers.

### Evaluation of CaDrA Reproducibility

Next, we sought to determine CaDrA’s reproducibility, and how this may be influenced by the statistical significance of the returned meta-feature (as determined by permutation *p*-value). To do so, we implemented a sub-sampling procedure and applied it to the search for YAP/TAZ activity drivers in TCGA BRCAs. Specifically, the original meta-feature returned by the search on the full dataset, and the meta-feature returned when performing the same search on a random subset (80%) of samples were compared by the Jaccard (*J*) index (see section “Methods”). We performed this sub-sampling search procedure both with respect to the original sample ranking (Figure 5A), and with respect to a permuted sample ranking (*n* = 100 iterations each). Comparison of the resulting *J* index distributions yielded a significantly higher reproducibility of results when sub-sampling from the original sample ranking, than from the randomly permuted one (Wilcox *P* < 0.0001; Figure 5D). These results support the conclusion that the CaDrA-based significance testing is a strong predictor of a search result reproducibility, and a rigorous criterion to discriminate between true and false positives.

**FIGURE 6:**
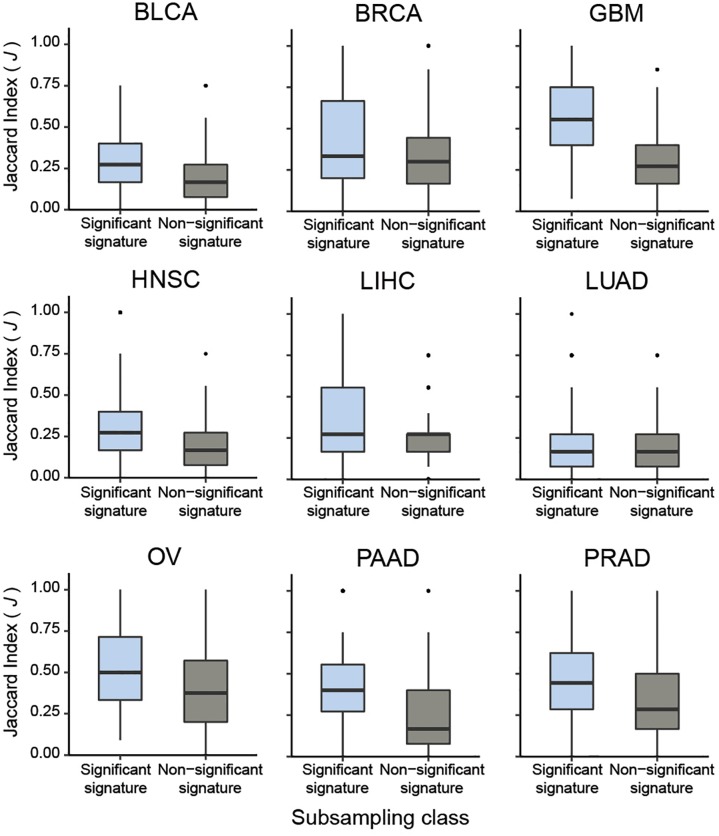
Pan-cancer sub-sampling analysis confirms agreement between CaDrA search significance and reproducibility of identified meta-features. CaDrA was applied to search for genomic alterations associated with elevated protein expression for all proteins profiled using RPPAs, for nine different cancer types in TCGA. Reproducibility by sub-sampling was then assessed for the top 5 significant (CaDrA *P* ≤ 0.05), and 5 non-significant (CaDrA *P* > 0.05) protein queries (see text). Consistency of CaDrA results was computed by the Jaccard (*J*) index of the returned meta-feature obtained with and without sub-sampling for each iteration, with the *J* indices pooled for the 5 significant and non-significant results, respectively. Box plots highlight a significantly higher *J* index coefficient among the significant protein queries compared to the non-significant queries across all cancer types investigated (Wilcox FDR < 0.001).

To systematically validate this conclusion, we extended the sub-sampling analysis to CaDrA queries of protein expression profiles across the nine different cancer types previously described. Briefly, for each cancer type we assessed whether the meta-features corresponding to the top five most-significant CaDrA protein queries (CaDrA *P* ≤ 0.05) were more reproducible than those corresponding to a randomly selected subset of five non-significant protein queries (CaDrA *P* > 0.05). To this end, the *J* index distribution obtained upon sub-sampling from the significant queries (*n* = 100 iterations each) was compared to the equivalent distribution from the non-significant queries, and a significantly higher reproducibility of the former was observed in all nine cancer types tested (Wilcox FDR < 0.001; Figure 6).

Taken together, these results show that CaDrA-based significance testing is a strong predictor of a search result reproducibility. Most importantly, it provides for a statistically rigorous decision rule, which would not be available based on the sub-sampling results alone.

## Discussion

Identifying (epi)genetic drivers of molecular readouts is of fundamental importance to determining alternative mechanisms influencing the phenotype in question. Existing methods attempting to extract functionally relevant sets of genomic alterations associated with a given context either do not support the analysis of data beyond somatic mutations, do not incorporate multiple feature scoring functions and search modes, or do not implement rigorous statistical significance testing of the obtained results. Importantly, a computational framework package bundling all of these features does not exist, and can significantly help identify novel drivers of signature activity.

Here, we presented CaDrA as a tool that determines the subset of queried binary features most associated with a phenotypic signature of interest by specifically exploiting a stepwise heuristic search method. CaDrA was applied to identify both known and novel genomic drivers of sample signature activity, comprising drug sensitivity, protein expression and gene set activity estimates, using publicly available multi-omics datasets from cancer cell lines and primary tumors. Querying CCLE data for features associated with increased sensitivity to Mek/Raf inhibitors, CaDra recovered known driver mutations in oncogenes known to be gate-keepers of MEK pathway activity, including *NRAS* and *BRAF*. Importantly, BRAF^*V*600*E*^ mutations account for >90% of BRAF mutations and is generally found to be mutually exclusive to *NRAS* mutations (Sensi et al., 2006; Cantwell-Dorris et al., 2011), as also observed in the CCLE, highlighting CaDrA’s ability to identify features exhibiting mutual exclusivity. Further, the large-scale investigation of expression profiles of annotated hallmark proteins in tumors from nine different cancer types in TCGA confirmed CaDrA’s ability to systematically identify known mutations of oncogenes and tumor suppressor genes in human cancers, as defined in the COSMIC database.

Through our extensive evaluation on simulated data, we were able to highlight CaDrA’s high sensitivity for mid-to-large sized datasets (*N* > 90), and high specificity for all sample sizes considered. Importantly, multi-omics datasets produced by networks such as CCLE and TCGA, also presented in this study, are well above this sample size limit. CaDrA’s specificity was further evident when querying genetic drivers of increased sensitivity to treatment with PLX4720, a potent and selective inhibitor designed to preferentially inhibit active B-Raf protein bearing the V600E allele (Tsai et al., 2008). In this scenario, the search process correctly identified the BRAF^*V*600*E*^ mutation as the sole feature associated with elevated sensitivity to treatment, in agreement with the known specificity of the small molecule inhibitor, with the feature association being highly statistically significant. It is important to emphasize that the evaluation of CaDrA’s sensitivity and specificity crucially relied on the statistical testing procedure we defined, a feature missing in most of the other existing methods.

We were also able to demonstrate the utility of our framework in the discovery of novel drivers in human breast cancers. Specifically, we asked whether there were genomic alterations associated with elevated activity of Hippo pathway co-activators YAP/TAZ, known to control pro-tumorigenic signals in multiple cancer types (Hiemer et al., 2015; Moroishi et al., 2015; Zanconato et al., 2016). The mechanisms contributing to dysregulated YAP/TAZ activity in cancer remain poorly understood. To date, very few genomic alterations have been associated with driving tumorigenic YAP/TAZ activity (Harvey et al., 2013). Our CaDrA search with respect to a sample ranking of decreasing YAP/TAZ activity, as measured by the coordinated expression of YAP/TAZ-activated genes, yielded a meta-feature consisting of chromosomal deletions of 5q21.3 and 20p13, and mutations in the *RELN*. Subsequent functional validation by knockdown of select targets, namely RELN and RBCK1, in HS578T BRCA cells exhibiting low YAP/TAZ-activity resulted in a significant increase in the expression of canonical YAP/TAZ targets CTGF and CYR61. These results confirmed the selected targets’ involvement in the regulation of YAP/TAZ-mediated activity, and the capability of CaDrA to identify new drivers of pathway activity. Importantly, this case study highlights the capability of the method to integrate information, and discover targets pertaining to multiple DNA alteration types.

A sub-sampling-based assessment of CaDrA’s results show that the ability to recover reproducible meta-features was higher for the true (significant) YAP/TAZ activity ranking, compared to a randomly permuted sample ranking. This sub-sampling procedure was independently assessed using a systematic pan-cancer comparison of reproducibility results from significant and non-significant protein queries, which revealed a significantly higher concordance of the former compared to the latter in all cases tested. Together, these results confirm the agreement between the estimated permutation *p*-values and the reproducibility of the meta-features identified by CaDrA, and emphasize the importance of our statistical testing procedure in supporting normative decision making.

Previously developed methods have indeed been shown to aid in the selection of functionally relevant genomic features in cancer (Ciriello et al., 2012; Vandin et al., 2012; Leiserson et al., 2013, 2015; Kim et al., 2016). However, CaDrA is to our knowledge the only method performing *rank-based* prediction in this context, which we believe is well-suited to: (i) model the noisy relationship between (epi)genetic alterations and a functional readout, and (ii) privilege the accurate prediction of highly ranked samples over lowly ranked samples, a desirable feature when modeling oncogenic activity. Furthermore, the framework as defined is flexible enough such that non-rank-based scoring functions can be easily incorporated. We emphasize that using rank-based scoring functions, while advantageous for the reasons mentioned, rely on accurate stratification of samples based on the dependent variable to yield concordant associations for a given biological question. Thus, the soundness of predictions is dependent on the quality of signatures used to query the target profile of interest.

The method that most-resembles CaDrA in its approach is REVEALER (Kim et al., 2016), an iterative search algorithm that functions in a similar fashion to CaDrA, while specifically seeking only those features that are mutually exclusive given the sample context. We note that a direct and rigorous comparison between CaDrA and REVEALER was not possible given the lack of a formal procedure to estimate statistical significance of results in the latter. We further emphasize that our tool defines a flexible framework capable of incorporating additional feature scoring functions, including the mutual information criterion implemented in REVEALER. Indeed, the incorporation of such scoring functions would benefit from the statistical significance estimation module built into CaDrA.

Current implementations of CaDrA and other similar methods are limited to the use of summarized input genomic features that are treated as binary events, denoting the presence or absence of a given mutation or SCNA in a sample. As we have demonstrated, this summarization approach is indeed sufficient to identifying genomic feature sets that may drive the target profile of interest. However, since different types of point mutations (missense, truncating, etc.) may impose differing functional impacts in oncogenes versus tumor suppressor genes, we surmise that these methods could be further improved by qualitatively differentiating between the different types of alterations being considered. One possibility would be to separate mutations by predicted gain or loss-of-function, as well as to distinguish between low (1) and high (≥2) DNA copy number gains or losses, although this may lead to excessive sparsity in the input matrix for low-frequency point mutations and SCNAs.

While our evaluations focused on somatic mutations and SCNAs, CaDrA’s search functionality can be applied to additional sequencing readouts capturing regulatory features, including and not limited to, DNA methylation and microRNA expression, albeit with proper discretization of these continuous features. A joint analysis of these additional data types might provide insight into epigenetic mechanisms that complement the assessed genetic features in driving phenotypic variation. Furthermore, we envision the adoption of CaDrA for the study of germ-line variation as well, thus contributing to move beyond the “one feature at a time” paradigm typical of GWAS studies, although issues of computational efficiency in that problem space will likely become more challenging.

## Conclusion

CaDrA enables the efficient identification of subsets of genomic features, including somatic mutations and SCNAs, as candidate drivers of a pre-defined phenotypic variable. Given the rapid rise in the availability of multi-omics datasets, as well as an increased need to interrogate targeted molecular readouts within these contexts, we believe that our methodology will accelerate feature prioritization for further follow-up and consideration, in turn aiding in the discovery of potential drivers of the phenotype of interest. Thus, we propose CaDrA as a tool for both targeted hypotheses testing, and novel hypothesis generation.

## Methods

### The CaDrA Algorithm

An overview of CaDrA’s workflow is summarized in Figure 1. CaDrA takes as input the sample ranking induced by a sample-specific measurement, a matrix of binary features (1/0 indicating the presence/absence of a given feature in a sample), and a scoring method specification to measure the significance of the concordance between the occurrence of alteration events and the defined sample ranking. The pre-defined sample ranking can be based on quantitative estimates of a gene expression, a signature or pathway activity, or other experimentally derived measurements. Each row in the matrix of binary features denotes the presence or absence of a somatic alteration (mutation, CNA, or other) in each of the samples in the ranked cohort. The score function is a measure of the *left-skewness* of a binary vector with respect to the sample ranking. The more the occurrences of an alteration are skewed toward higher rankings (i.e., the more the 1’s in the feature vector are skewed toward the left), the higher the score. The scores currently implemented are the KS test (default), and the Wilcoxon rank-sum test, but additional scoring functions can easily be added.

Given the sample ranking, the matrix of binary features, and the score of choice (KS or Wilcoxon), CaDrA implements a step-wise greedy search: it begins by first selecting the single feature that maximizes the score (Step 1; Figure 1). It then generates the union (logical OR) of this starting feature with every other remaining feature in the dataset and computes scores for the obtained ‘meta-features’ (Step 2; Figure 1); it selects a 2nd feature that, added to the first (as a union), maximally increases the score – which will then serve as the new top reference hit (Step 3; Figure 1). Repeating this process until no further improvement to the cumulative score can be attained, the search output is a set of features (i.e., a meta-feature) whose union has the (local) maximum skewness score with respect to the input sample ranking. The significance of a CaDrA search and its cumulative score are determined by generating an empirical null distribution of scores based on the exact same data and search parameters, but with randomly permuted sample rankings, providing a permutation *p*-value per search result. Since the CaDrA algorithm specifically returns feature-sets maximally left-skewed given the provided sample ranking variable, it can be applied to identify features that are either positively correlated or anti-correlated with the continuous variable of interest by ranking samples in decreasing or increasing order of that variable, respectively.

### CaDrA Features

#### Search Modes

CaDrA supports multiple search modalities: it allows for the selection of a user-specified feature from which to start the search (rather than selecting the feature with highest score as depicted in Step 1 of Figure 1); alternatively, since the greedy search is not guaranteed to find the global maximum, it also allows for a “top-N” search modality, whereby the search is started from each of the first N features (as measured by their individual skewness scores), and the result of the best search can be determined by selecting the set of features with the best cumulative score over the top-N runs.

#### Visualization of Search Results

For a given search, CaDrA outputs a set of features (meta-feature), which can be visualized as a ‘meta-plot’. This includes (panels from top to bottom): an area plot of the sample-specific measurements used to obtain the sample ranks; a color-coded matrix of all features in the meta-feature (in the step-wise order that they were added), one feature per row, with the corresponding union of the meta-feature (red) last; and a corresponding enrichment score (ES) plot below. Additionally, top-N search results can be visualized for overlapping features to evaluate robustness across different search starting points.

#### Parallelization Support

The generation of the empirical null distribution for significance testing is typically done for ≥500 iterations (i.e., permuted sample ranks). In order to speed up this potentially time-consuming task, CaDrA supports exploiting parallel computing with the help of the parallel R package functionality, should multiple compute cores be available to users.

#### Permutation Caching

Since the generation of the null distribution used for significance testing is a time-consuming step, and since the null distribution of scores depends solely on the feature dataset and the search parameters specified (scoring method, starting feature versus top-N search mode etc.), and not on the input sample ranking, we can implement cacheing of the null distribution corresponding to each dataset and search parameters. When submitting multiple subsequent queries (each with its own sample ranking) that utilize the same dataset and search criteria, CaDrA can then fetch the corresponding cached null distribution to generate permutation *p*-values almost instantaneously, avoiding the need for repetitive computation, thus significantly reducing overall query run time.

### Data Availability and Processing

CaDrA is freely available for download and use as a documented R package under the git repository https://github.com/montilab/CaDrA, and will further be deposited and maintained for future use under Bioconductor, including complete code and example use-cases.

DNA copy number (GISTIC2), mutation and RPPA data for TCGA analyses were obtained using Firehose v0.4.3 corresponding to the Jan 28th, 2016 (SCNA and somatic mutations) and Jul 15th, 2016 (RPPA) Firehose release. Somatic mutation data was processed at the gene level by assigning either 1 or 0 based on the presence or absence of any given mutation in that gene, respectively (excluding synonymous mutations). Annotated Level 3 RPPA data was used for all protein-related TCGA data queries. For pan-cancer analyses, these three data sets were obtained for nine cancer types, including bladder urothelial carcinoma (BLCA), breast invasive carcinomas (BRCA), glioblastoma multiforme (GBM), head and neck squamous cell carcinoma (HNSC), liver hepatocellular carcinoma (LIHC), lung adenocarcinoma (LUAD), ovarian serous cystadenocarcinoma (OV), pancreatic adenocarcinoma (PAAD), and prostate adenocarcinoma (PRAD). RNASeq version 2 data processed as Level 3 RSEM-normalized gene expression values corresponding to the Feb 4th, 2015 Firehose release was used for the TCGA BRCA analysis. CCLE genomic data were downloaded from https://portals.broadinstitute.org/ccle and processed as previously described (Kim et al., 2016). Somatic mutation binary calls per gene were used as is, and SCNA data was processed using GISTIC2 (Mermel et al., 2011) with all default parameters barring the confidence level, which was set to 99%. ActArea estimates pertaining to drug treatment sensitivity across CCLE samples was used as previously described (Barretina et al., 2012).

In all cases presented, SCNA and somatic mutation data were jointly analyzed as a single input dataset to CaDrA, thereby including samples for which both data were available. All input data to CaDrA were further pre-filtered so as to exclude alteration frequencies below 3% and above 60% to reduce feature sparsity and redundancy, respectively, across samples (CaDrA’s default feature pre-filtering settings).

### Simulated Data Generation

To evaluate both the sensitivity and specificity of CaDrA, we generated simulated data to represent cases where there was a mix of left-skewed (“true positive”) and randomly distributed (“null”) features, as well as cases where there were only null features. The left-skewness of a feature is a measure of its association with the sample ranking, since samples are sorted from left (high rank) to right (low rank). The design and parameter specification of the simulated data matrix is shown in Supplementary Figure S1. Each feature/row is a binary (0/1) vector, with 1 (0) in the *i*th position denoting the occurrence (non-occurrence) of the genetic event (e.g., SCNA or mutation) in the *i*th sample. This simulation of binary features relies on the following parameters:

*N*: Dataset sample size (number of columns in the matrix).
*n*: Total number of features in the dataset (number of rows in the matrix).
*p*: Number of true positive features generated per dataset [a positive feature is a feature whose distribution of events (i.e., the number of 1’s) is significantly associated with the sample ranking, i.e., left-skewed].
*f*: Left-skew proportion. The proportion of samples that are *cumulatively* left-skewed in the sample ranking.
λ: The mean (and variance) of the Poisson distribution from which the number of events in the null features is sampled. This is equal to the number of 1’s per skewed positive feature. A Poisson distribution is used so that we can partially control (through the mean) the number of 1’s in a null feature, which are then uniformly distributed across samples (see description of Null feature generation below).

The resulting simulated binary data matrix will consist of two main types of features:

*True Positive (TP) Features:* A total of *p* TP features are generated. Events (i.e., 1’s) are assigned to the TP features in a mutually exclusive fashion, with each of these features having (*f × N*)*/p* entries set to 1, with their cumulative OR yielding an N-sized vector with the left-most *f × N* entries set to 1’s. For example, if we generate data for 100 samples and 5 positive features, with the left-skew proportion set to 0.5, each non-overlapping feature will have 10 among the 50 left-most entries (columns) set to 1, such that the union (logical OR) of the 5 features will have 1’s in the first 50 entries.
*Null Features:* Null features are generated for a total of (*n–p)* features. To generate these features, we sample the number of 1’s per null feature based on a Poisson distribution with mean parameter λ = (*f × N*)/*p*. In this fashion, the number of 1’s in the null features will have a distribution centered on the corresponding number for the TP features. For instance, if we generate data for 100 samples and 5 TP features with left-skew proportion *f* = 0.5, then each of the TP features will have ten 1’s, and each of the remaining 995 null features will have a number of 1’s sampled from Poisson (λ = 10), uniformly distributed over the *N* samples.

A schematic representation of this data, along with the parameters that define its composition is shown in Supplementary Figure S1.

### Evaluation of CaDrA Performance on Simulated Data

Evaluation of CaDrA performance was performed considering two main scenarios: (a) True positive datasets: Data containing both true positive and null features (where the sensitivity of CaDrA is tested); and (b) Null datasets: Data containing only null features (where the specificity of CaDrA is tested), with the following parameter specifications for data generation:

*N* = {50, 60, 70, 80, 90, 100, 250, and 500}
*n* = 1000
*p* = 5
*f* = 0.5

CaDrA was run using default input parameters, returning a meta-feature which had the best score, along with a permutation *p*-value based on the empirical null search distribution (Supplementary Figure S2). These results were then used to determine performance estimates for different sample sizes, composition (i.e., distribution of TP versus null features per returned meta-feature), size (i.e., the number of features within the returned meta-feature) and statistical significance of the returned meta-features. Mean TPR percentages shown in Table 1 are a result of weight-averaging TPRs corresponding to different number of true positive features per meta-feature, weighted by the total searches returning such meta-features (gray circles Figure 2C). Mean FPR percentages shown in Table 1 are a result of weight-averaging FPRs corresponding to different meta-feature sizes, weighted by the total searches returning such meta-features (gray circles Figure 2D).

### COSMIC Enrichment Analyses

For enrichment analyses, RPPA protein data for the nine cancer types (see section “Data Availability and Processing”) was first restricted to those proteins representing hallmark oncogene or tumor suppressor genes included in the COSMIC v84 database (*n* = 57)^1^ (Forbes et al., 2017). For each cancer type, a CaDrA query was then performed with respect to the protein expression-induced sample ranking, using somatic mutation and copy number alteration data as input features, in order to search for features associated with elevated protein expression of each of the hallmark proteins queried. The features selected thereof were then pooled across all queries, and the resulting gene list tested for significant enrichment (based on the hyper-geometric distribution) with respect to a set of annotated oncogenes and tumor suppressor genes in COSMIC (*n* = 554), compared to the pooled list of non-selected features.

### Sub-Sampling Analyses

For all sub-sampling analyses presented, CaDrA was run after sub-sampling 80% of the original data, with consistency of CaDrA results computed as the Jaccard (*J*) index of the returned meta-feature obtained with and without sub-sampling (repeated for *n* = 100 independent sub-sampling iterations). To assess reproducibility of drivers associated with YAP/TAZ activity, the search was repeated by either preserving the observed ranking (decreasing YAP/TAZ activity), or by taking a permuted ranking. *J* indices were then compared between the original and permuted ranking cases using a Wilcox rank sum test. For the pan-cancer protein query analysis, all available proteins profiled as part of the RPPA data were used, with *J* indices similarly computed for the top 5 protein queries that yielded significant meta-features (*P* ≤ 0.05), and 5 queries randomly selected from the non-significant list (*P* > 0.05) in each cancer type. *J* indices were then pooled for the five significant, and non-significant results, respectively, and compared using a Wilcox rank sum test. FDR correction was used for all pan-cancer analyses tests of significance.

### YAP/TAZ Signature Projection and Assessment in TCGA BRCAs

A signature comprising YAP/TAZ-activating genes (*n* = 717) in MDA-MB-231 cells was obtained based on a previous study (Enzo et al., 2015). The TCGA BRCA RNASeq data (*n* = 1,186 samples) was projected onto the signature genes and per-sample estimates of YAP/TAZ activity were derived using ASSIGN (Shen et al., 2015), which was then used as a continuous ranking variable with CaDrA. The association of YAP/TAZ activity with the CaDrA-derived meta-feature, and with BRCA subtype (i.e., TN status) was determined using a linear regression model.

### Cell Culture, siRNA Knockdown and qRT-PCR

HS578T BRCA cells were purchased from ATCC and cultured using media and conditions suggested by ATCC. For RNA interference, cells were transfected using RNAiMAX (Thermo Fisher) with control siRNA (Qiagen, 1027310) or an equal molar mixture of siRNA targeting RELN (Sigma), RBCK1 (Sigma), or TAZ and YAP (Hiemer et al., 2014). 48 h post transfection, RNA was extracted from cells using RNeasy kit (Qiagen) and the synthesis of cDNA was performed as previously described (Hiemer et al., 2014). Quantitative real-time PCR (qRT-PCR) was performed using Taqman Universal master mix II (Thermo Fisher) and measured on ViiA 7 real-time PCR system. Taqman probes used included those recognizing CTGF (Thermo Fisher Hs00170014_m1), CYR61 (Thermo Fisher Hs00155479_m1), RELN (Thermo Fisher Hs01022646_m1), RBCK1 (Thermo Fisher Hs00934608_m1), WWTR1 (Thermo Fisher Hs01086149_m1), and YAP (Thermo Fisher Hs00902712_g1) and GAPDH (Thermo Fisher 4326317E). Expression levels of each gene were calculated using the ΔΔCt method and normalized to GAPDH. Knockdown efficiency of YAP, TAZ, RELN, and RBCK1 was verified for each experiment. Mean transcriptional knockdown of YAP, TAZ, and RBCK in HS578T cells was >80%. Basal RELN levels in HS578T cells were low, and relative knockdown in these cells was 28.3% (±14.1). Data from qRT-PCR experiments are shown as mean ± S.D., with each knockdown compared with respect to the scrambled siRNA control (siCtl) using an unpaired, two-tailed Student’s *t*-test.

### CaDrA Search Parameters

For evaluation using genomic data, CaDrA was run in the top-N mode using the default of *N* = 7, choosing the best resulting meta-feature (see section “Methods”; CaDrA features: Search modes). For evaluation of simulated data, only the top-scoring feature was considered as a starting feature per search run (i.e., *N* = 1). The “ks” method was chosen for evaluating skewness of features at each step in all cases presented. All other default input search parameters were used for all cases presented.

## Availability of Data and Material

The datasets generated and/or analyzed during the current study are available in the TCGA repository (https://tcga-data.nci.nih.gov/docs/publications/tcga), and CCLE repository (https://portals.broadinstitute.org/ccle), and are available from the corresponding author on reasonable request.

## Author Contributions

VK developed the R package and conducted the analyses. VK and SM wrote the manuscript, with input from PS and XV. JK performed the siRNA and qRT-PCR experiments. LZ assisted in obtaining the gene expression signature for TCGA data projection. PS assisted in the evaluation of CaDrA on simulated data. SM and VK designed the CaDrA framework and features, and interpreted the results. XV designed the experimental validation of novel candidate drivers, and interpreted the results thereof. All authors read and approved the final manuscript.

## Conflict of Interest Statement

The authors declare that the research was conducted in the absence of any commercial or financial relationships that could be construed as a potential conflict of interest.

## Footnotes

1 https://cancer.sanger.ac.uk/census

## Abbreviations

BRCA: breast carcinomas
CaDrA: candidate driver analysis
CCLE: Cancer Cell Line Encyclopedia
COSMIC: Catalogue of Somatic Mutations in Cancer
FDR: false discovery rate
FPR: false positive rate
KS: Kolmogorov–Smirnov
qRT-PCR: quantitative real-time polymerase chain reaction
RPPA: reverse phase protein array
SCNA: somatic copy number alteration
TCGA: The Cancer Genome Atlas
TN: triple-negative
TPR: true positive rate
