# Supplementary material for: CaDrA: A Computational Framework for Performing Candidate Driver Analyses Using Genomic Features
Source: Front Genet. 2019 Feb 19;10:121. doi: 10.3389/fgene.2019.00121 (PMC6390206; doi:10.3389/fgene.2019.00121)
Supplement: Supplementary file 1 [file Data_Sheet_1.PDF]

Supplemental Information pertaining to *Kartha et al. CaDrA: A computational framework for performing candidate driver analyses using genomic features*

Supplemental Figures

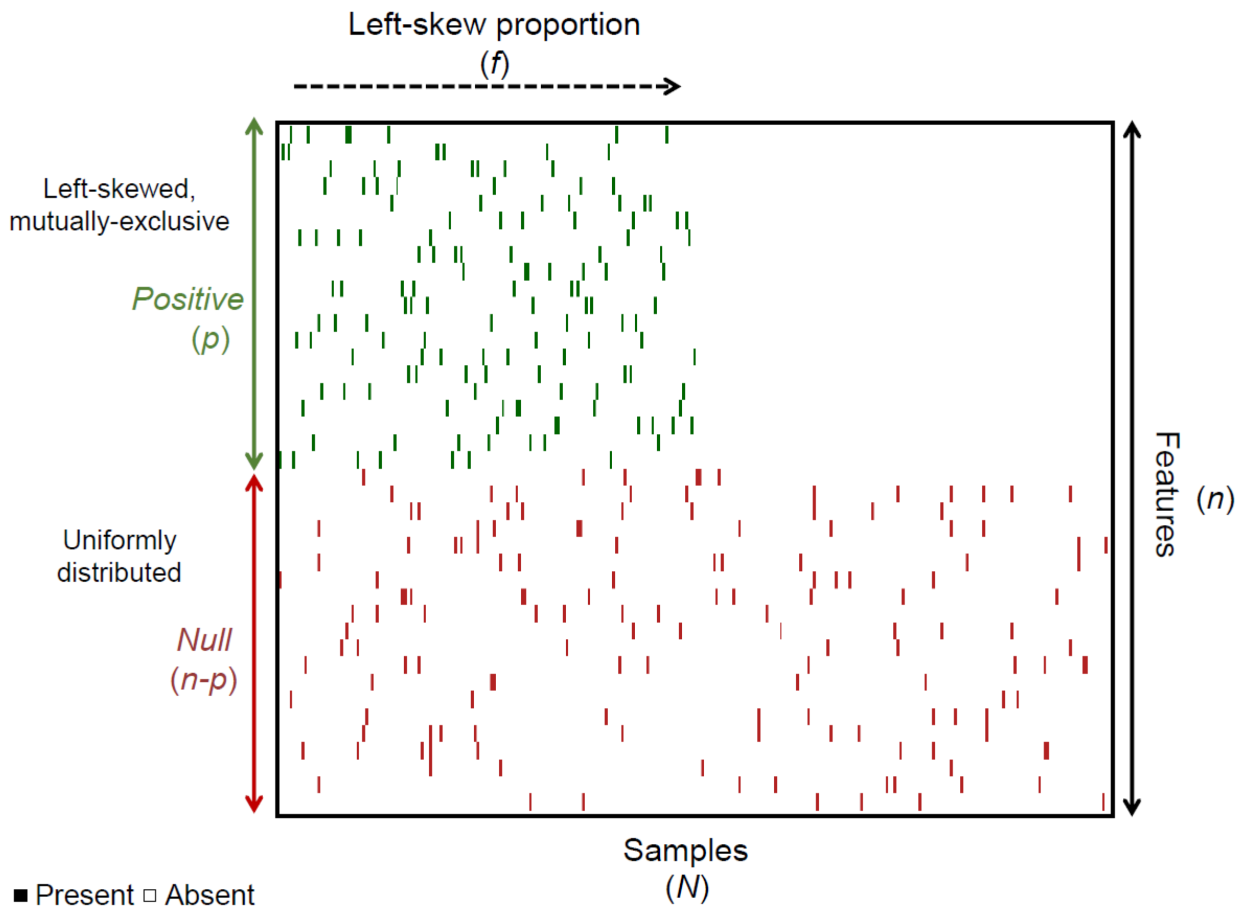

Figure S1. Schematic representation of a simulated dataset used for CaDrA performance evaluation. A typical binary matrix highlighting the nature of the left-skewed (positive) features and the uniformly distributed (null) features is shown. CaDrA was evaluated based on simulated datasets containing either only null features, or those containing both positive and null features (as shown in schematic) to determine specificity and sensitivity estimates across search results, respectively.

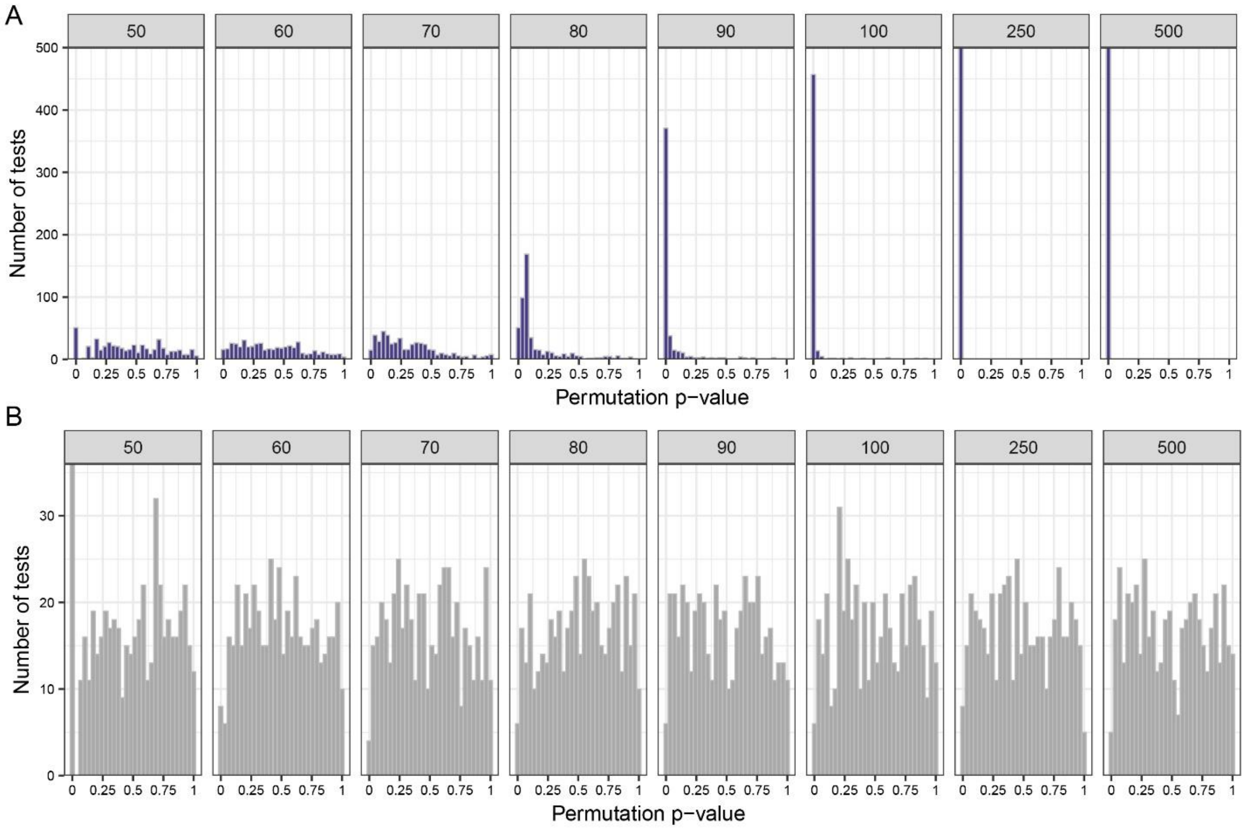

Figure S2. Distribution of search permutation p-values across CaDrA search runs using simulated data for different sample sizes (*n*=500 simulations per sample size) in the positive (**A**) and null (**B**) simulated datasets.

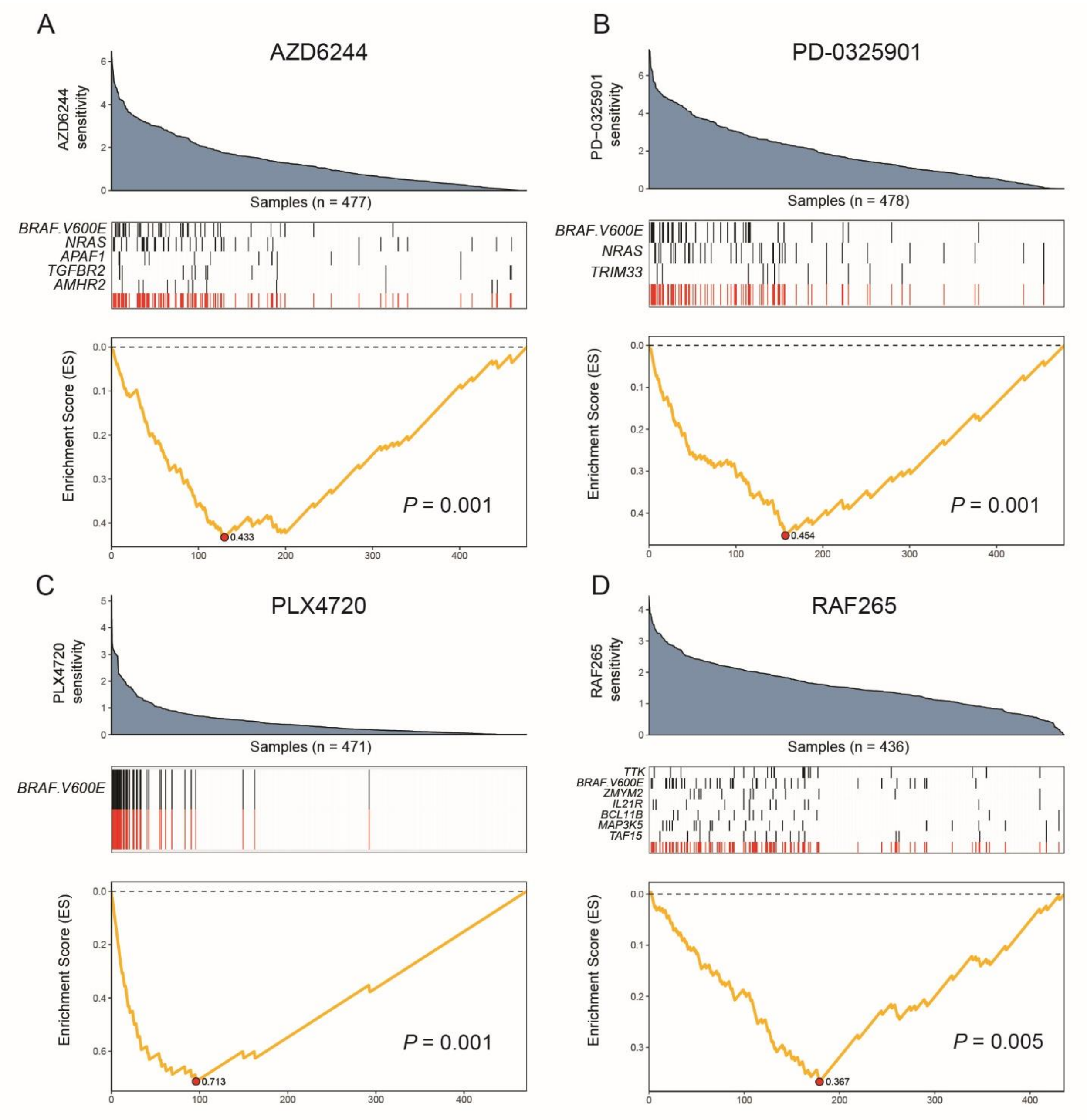

Figure S3. Candidate driver analysis hits associated with elevated sensitivity to Mek and Raf inhibition in cancer cell lines. CaDrA was run using ActArea drug sensitivity measurements pertaining to AZD6244 (**A**), PD-0325901 (**B**), PLX4720 (**C**) and RAF265 (**D**) treatment as a sample ranking variable, and somatic mutation and copy number alteration data as binary features from CCLE. The top hits (meta-feature) identified in each case are shown (also summarized in Table 2).

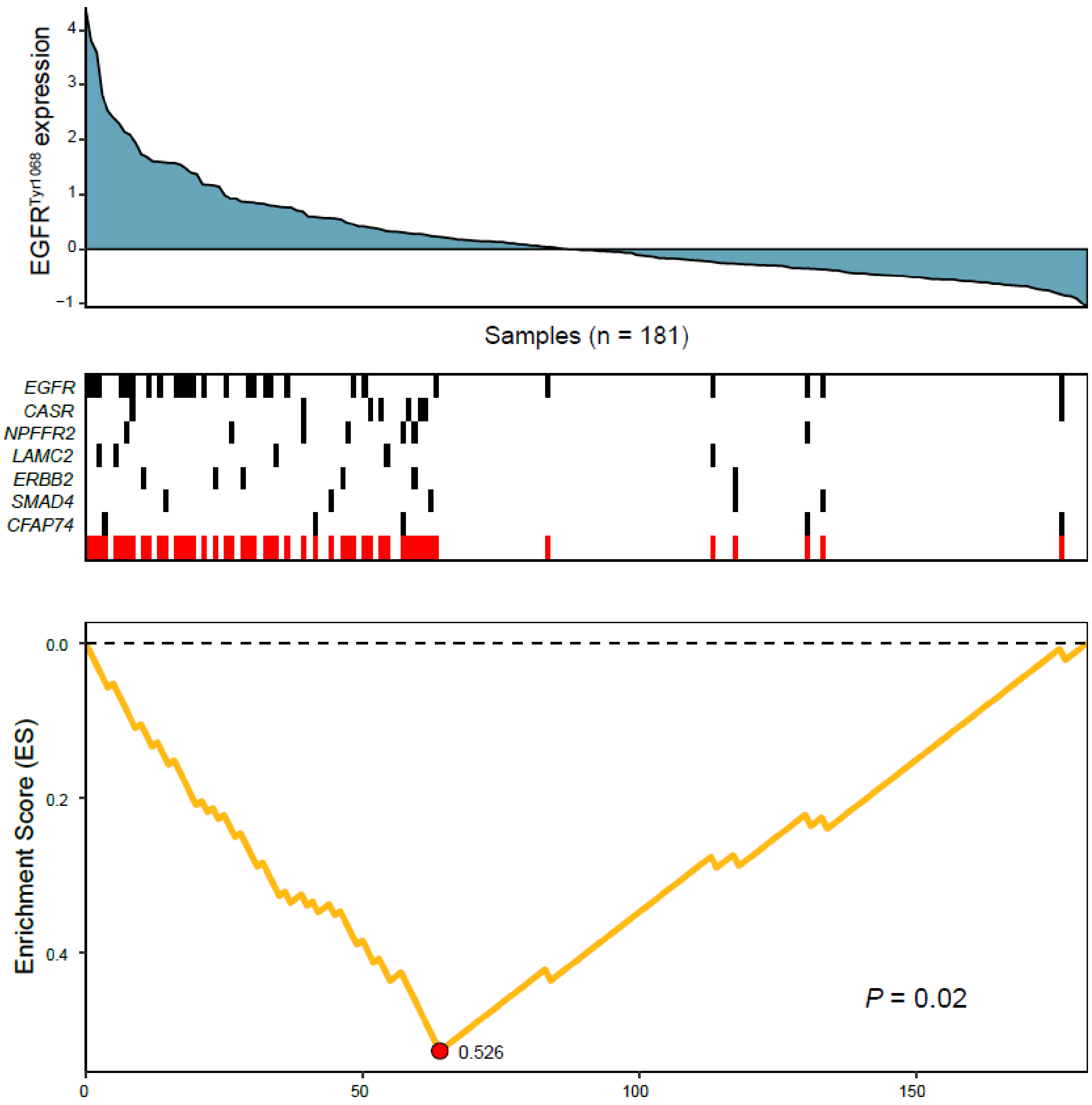

Figure S4. Seeded search for drivers of increased EGFR protein expression in TCGA lung adenocarcinoma (LUAD) samples identifies additional mutations in EGFR pathway-associated oncogenes. TCGA LUAD samples (*n*=181) were first ranked by decreasing phosphorylated EGFR ($EGFR^{Tyr1068}$) protein expression levels. CaDrA was then run specifically seeding the search with mutations in EGFR (starting feature) to look for additional features that, together, are most-associated with increased $EGFR^{Tyr1068}$ expression. The resulting meta-feature is shown, and includes mutations in known EGFR-associated genes *SMAD4* and *ERBB2*. The union of the identified features (red track) and the corresponding running enrichment score (ES) is also shown

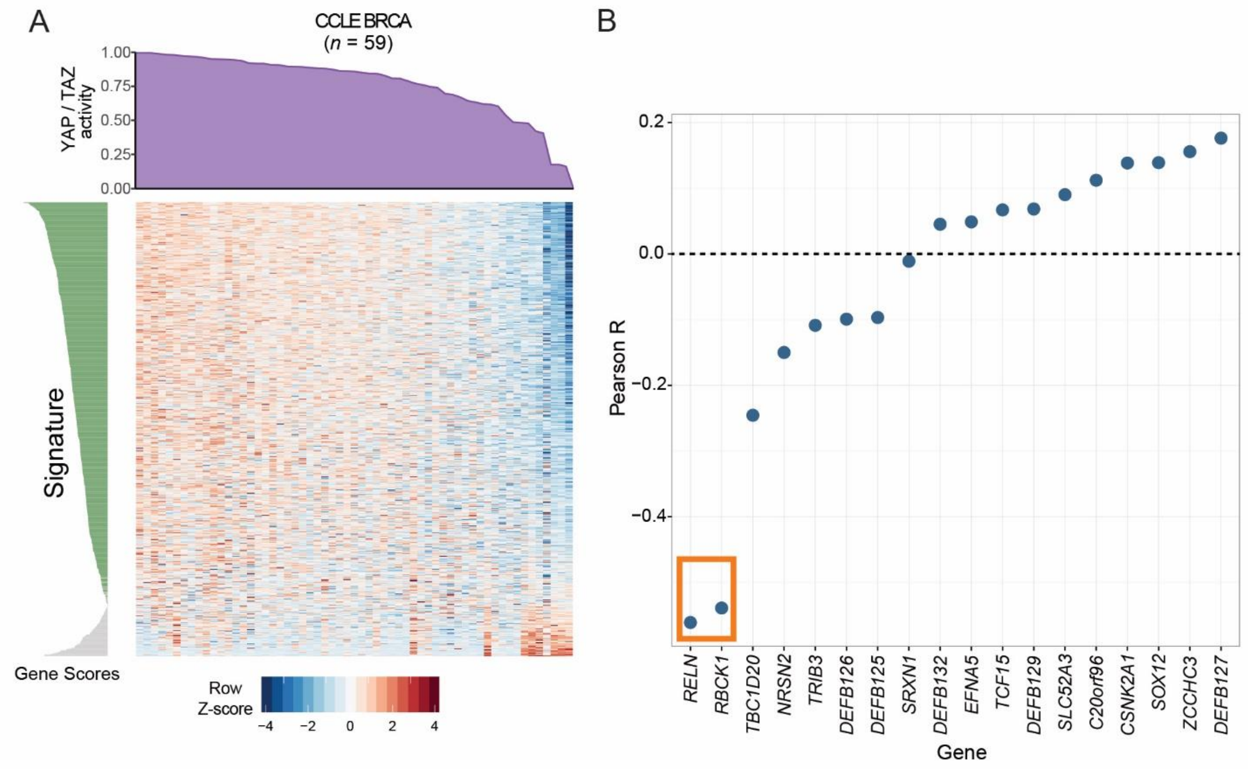

Figure S5. Interrogation of CaDrA hits associated with increased TCGA BRCA YAP/TAZ activity in CCLE BRCA cell lines. (**A)** Projection of CCLE BRCA cells (*n*=59) in the space of MDA-MB-231-derived YAP/TAZ-activating genes yielding activity scores per cell line (purple area plot) **(B)** Correlation of the derived YAP/TAZ activity scores with expression levels of genes identified by CaDrA to be associated with elevated YAP/TAZ activity (Table S1). *RELN* and *RBCK1* show relatively higher anti-correlation between YAP/TAZ activity and their mRNA expression levels, and were selected for knockdown and qRT-PCR validation *in vitro*. Only genes with mRNA expression profiles in CCLE are shown.

Supplemental Tables

Cancer Type	Sample size	FDR q-value
BLCA	116	0.029
BRCA	783	1.53e-04
GBM	138	1.53e-04
HNSC	200	2.63e-06
LIHC	158	1.21e-06
LUAD	181	0.022
OV	208	1.53e-04
PAAD	100	5.57e-04
PRAD	248	2.63e-06

Table S1. FDR q-values of gene set hyper-enrichment for TCGA pan-cancer COSMIC analysis. Sample sizes indicate number of samples with RPPA, GISTIC2 and mutation data available (see “Data availability and processing” in manuscript methods for details)

<table>
<tr><th>CaDrA hits</th><th>Genes in region</th><th>P-value</th></tr>
<tr><td>Del5q21.3</td><td>RN7SL782P, SNORA31, EFNA5</td><td rowspan="3">0.001</td></tr>
<tr><td>Del20p13</td><td>TCF15, CSNK2A1, SOX12, RBCK1, TRIB3, NRSN2, ZCCHC3, SCRT2, SLC52A3, TBC1D20, C20orf96, SRXN1, DEFB125, DEFB126, DEFB127, DEFB128, DEFB129, DEFB132</td></tr>
<tr><td>RELN</td><td>N/A</td></tr>
</table>

Table S2. Candidate driver analysis hits associated with elevated YAP/TAZ activity in TCGA BRCA samples. Genes within each chromosomal deletion peak are highlighted.
